# Biochar mitigates polyethylene and oxytetracycline stress in *Amaranthus tricolor* by improving soil properties, reducing oxidative damage, and moderating CO_2_ emissions

**DOI:** 10.3389/fpls.2026.1829340

**Published:** 2026-06-29

**Authors:** Ismail Khan, Muhammad Tariq, Asma Mehmood, Muhammad Sadiq, Faming Wang, Bi Zou, Faisal Nadeem, Abdul Rehman, Beena Saeed, Ping Zhuang

**Affiliations:** 1 Guangdong Provincial Key Laboratory of Applied Botany, South China Botanical Garden, Xiaoliang Research Station for Tropical Coastal Ecosystems, Key Laboratory of Vegetation Restoration and Management of Degraded Ecosystems, and the Chinese Academy of Sciences (CAS) Engineering Laboratory for Ecological Restoration of Island and Coastal Ecosystems, South China Botanical Garden, Chinese Academy of Sciences, Guangzhou, China; 2 School of the Environment and Safety Engineering, Jiangsu University, Zhenjiang, China; 3 Department of Agronomy, University of Agriculture, D.I. Khan (KPK), Dera Ismail Khan, Pakistan; 4 Faculty of Agriculture and Environment, The Islamia University of Bahawalpur, Bahawalpur, Pakistan; 5 Agronomy, Department of Agriculture, The University of Swabi, Anbar, Pakistan

**Keywords:** antibiotics, biochar, microplastics, plant growth, soil nutrients

## Abstract

Microplastics (MPs) and antibiotics (ATs) frequently co-occur in agricultural soils, but their combined impacts on soil–plant systems and potential remediation options remain unclear. This 60-day pot experiment tested whether maize biochar (20 t ha^-1^) can alleviate the effects of polyethylene (PE, 10 g kg^-1^) and oxytetracycline (OTC, 50 mg kg^-1^) on soil properties, antioxidant enzyme activities, and A. tricolor growth. Co-exposure to PE and OTC significantly reduced soil nutrient availability, suppressed root development by 23%, and decreased aboveground biomass by 45% relative to the control, while pollutant treatments enhanced antioxidant enzyme activities, indicating induced oxidative stress rather than improved defense alone. Biochar application increased soil pH, cation exchange capacity, and nitrogen availability (NO_3_^-^-N and NH_4_^+^-N by 22% and 19%, respectively), and partially mitigated contaminant-induced declines in photosynthesis, root traits, and biomass. Consistent with the CO_2_ measurements, biochar alone elevated short-term soil CO_2_ emissions by 49% and 27% at 30 and 60 days, respectively, whereas PE+OTC reduced CO_2_ fluxes by 55% and 29%, with biochar partly offsetting this suppression rather than uniformly lowering CO_2_. These findings suggest that corn straw biochar can alleviate phytotoxic effects of PE-OTC co-contamination and support crop production by improving soil nutrient status and moderating oxidative stress responses, while simultaneously altering soil carbon dynamics. From an applied perspective, biochar shows promise for managing co-contaminated vegetable soils, but future work should resolve underlying mechanisms especially pollutant bioavailability, microbial processes, and antibiotic resistance genes to guide safe, field-scale implementation.

## 1 Introduction

Antibiotic residues and microplastics (MPs) are emerging co-contaminants in agricultural soils, posing risks to soil health, crop productivity, and antibiotic resistance (Liao et al., 2025; Carter et al., 2026). The rapid increase in antibiotic resistance is a major public health concern (Fu et al., 2021). In agriculture, extensive antibiotic use in livestock and aquaculture leads to their release into soils via manure and wastewater, where they can persist and disrupt soil biota and plant performance (Fang et al., 2023). Oxytetracycline (OTC), widely used in veterinary and horticultural practices (Dréano et al., 2022), strongly sorbs to soil and can remain for long periods, threatening long-term soil function and food security (Liao et al., 2025). These risks are particularly critical for intensively managed leafy vegetables, where short growth cycles and high input use can enhance exposure of edible biomass to soil-borne contaminants (Roy et al., 2024).

In parallel, MPs (<5 mm) have become pervasive in croplands through plastic mulching, manure and sludge application, and irrigation (Wang et al., 2024c). Farmland MPs loads can exceed those in oceans (Huang et al., 2020; Zhao et al., 2026), with polyethylene (PE) and polypropylene (PP) dominating (Pinto-Poblete et al., 2023). As a result, MPs alter soil structure, water dynamics, nutrient cycling, and microbial communities (Khan et al., 2024a; Afzal et al., 2025b; Wang et al., 2026). These alterations, in turn, directly impact plant growth and performance. Concurrently, MPs-induced changes in soil properties also affect the retention, transport, and ecological impact of co-existing contaminants (Li et al., 2023). For instance, MPs and antibiotics frequently co-occur and are significantly correlated in manured farm soils, where their combined presence drives antibiotic resistance gene (ARG) dynamics (Wang et al., 2024b). PE and other polymers can adsorb OTC, reducing its mobility while increasing its bioavailability, thereby promoting ARG dissemination (Khan et al., 2024b; Yang et al., 2025). Furthermore, Co-contamination by PE and OTC can also disturb nutrient cycling and soil organic carbon (SOC) mineralization, with consequences for CO_2_ emissions (Khan et al., 2024b; Li et al., 2024). Despite this, most studies have addressed PE or OTC separately (Khan et al., 2024a; Shen et al., 2024; Zhang et al., 2024), and mechanistic evidence for their combined effects on soil-plant systems and greenhouse gas (GHG) fluxes critically limited. Notably, evidence on how such co-contamination affects nutrient-dense leafy vegetables, such as *Amaranthus tricolor*, is particularly scarce, despite their widespread consumption and economic importance (Roy et al., 2024).

Biochar is increasingly proposed to mitigate such emerging contamination while improving soil quality. Derived from biomass pyrolysis, biochar has high carbon content, porosity, and abundant functional groups, enhancing nutrient retention and contaminant sorption (Al Masud et al., 2023). Engineered and modified biochar can further enhance adsorption of organic pollutants, including antibiotics, and support rhizoremediation (Mustafa et al., 2026). Recent work shows that biochar can alleviate MPs impacts on soil properties, microbial communities, ARGs, and plant growth, and can reduce risks in co-contaminated systems (Cheng et al., 2025; Mingwei et al., 2026). Consequently, biochar is increasingly considered a promising strategy for the remediation of contaminated soils while simultaneously improving soil physicochemical properties. Nevertheless, the interactive effects of biochar with combined PE-OTC contamination on soil properties, plant performance, and CO_2_ emissions remain poorly constrained, particularly under biochar application rates consistent with practical field recommendations. This knowledge gap limits the development of biochar-based management strategies to mitigate risks associated with co-contamination by PE-OTC in vegetable cropping systems.

To address these gaps, the present study investigated the individual and combined effects of PE and OTC, and their interaction with biochar, on soil physicochemical properties, CO_2_ emissions, and the growth performance the leafy vegetable *Amaranthus tricolor*. This species was chosen as a representative, nutrient-dense C_4_ leafy vegetable widely cultivated and consumed in tropical and subtropical regions, with documented tolerance to abiotic stresses and high contents of vitamins, minerals, and antioxidant phytochemicals (Roy et al., 2024). Its short growth cycle and high biomass turnover make it a sensitive model for detecting sub-chronic soil contamination effects in intensively managed systems. It is commonly grown in intensively managed fields where plastic mulching and veterinary antibiotic inputs are frequent, and its short growth cycle makes it suitable for detecting sub-chronic stress responses. The PE and OTC concentrations, as well as the biochar application rate, were chosen to fall within or slightly above reported ranges in contaminated agricultural soils (Li et al., 2023; Tang et al., 2023), and recommended biochar doses (Khan et al., 2021), thereby ensuring both environmental relevance and detectable biological responses. Our study specifically focuses on the “PE + OTC co-contamination × biochar” interaction as a novel perspective to evaluate how biochar may modulate the joint impacts of PE and OTC on soil-plant-atmosphere processes. We tested the following hypotheses: (i) individual and combined exposure to PE and OTC would significantly alter soil physicochemical properties, CO_2_ emissions, and A. tricolor growth; (ii) co-exposure to PE and OTC would exert more pronounced adverse effects on soil-plant systems than single-contaminant exposure; and (iii) biochar amendment, at an agronomically realistic dose, would partially mitigate the negative impacts of PE and OTC on soil properties, CO_2_ emissions, and plant performance by altering contaminant bioavailability and the soil microenvironment.

## 2 Materials and methods

### 2.1 Materials preparation and characterization

*Amaranth tricolor* L. seeds were obtained from the Dezhou Degao Vegetable Seedling Research Institute, Shandong Province, China. For the pot experiment, the test soil was collected from an uncontaminated green area on the South China Botanical Garden, Chinese Academy of Sciences (CAS), Guangzhou, China. The collected soil was air-dried, homogenized, and sieved through a 2 mm mesh to remove debris; its physicochemical properties were then analyzed (**Supplementary Table 1**). The antibiotic used was Oxytetracycline (OTC) (C_22_H_24_N_2_O_9_; 98% purity, CAS: 79-57-2), selected due to its widespread presence in agricultural soils. Stock solutions of OTC were prepared in methanol, diluted with deionized water, and thoroughly mixed with soil to achieve the target concentration. The OTC was procured from Shanghai Aladdin Biochemical Technology, P.R. China, with a purity of 98%. OTC is commonly used in veterinary and agricultural systems, making it relevant for assessing its impacts on soil health and plant growth. Commercially available polyethylene (PE) (100 µm-200 µm, irregular shape) was purchased from Zhangmutou Huahuang Plastic Material Company in Dongguan, China. PE was applied at a concentration of 1% (w/w) in the soil. To remove residual solvents, PE particles were rinsed with 70% ethanol and deionized water, then air-dried in a fume hood for 48 h.

Biochar was produced from corn straw through pyrolysis at 600 °C. The process involved a heating rate of 10 °C/min and a two-hour retention time under a nitrogen (N_2_) atmosphere to ensure an oxygen-limited environment. All materials were characterized as follows: surface functional groups of PE and biochar were analyzed using Fourier Transform Infrared Spectroscopy (FTIR) (**Supplementary Figure 1A**); crystalline structure was assessed via X-ray diffraction (XRD) (**Supplementary Figure 1B**); elemental composition was determined using Energy-Dispersive X-ray Spectroscopy (EDS); and surface morphology was examined using scanning electron microscopy (SEM) (**Supplementary Figure 2**).

### 2.2 Experimental design and growth conditions

A pot experiment was conducted in 2024 at the South China Botanical Garden (CAS), Guangzhou, China, (23°10’ N, 113°21’ E), following a two-factor, completely randomized design. Factor A contains biochar amendments, i.e., Control (BC0, no biochar) and biochar applied (BC1, 20 t ha^-1^). Whereas factor B had four levels of soil contaminants: Control (CK), OTC (50 mg kg^-1^ soil), PE (10 g kg^-1^ soil), and their combined application (OTC+PE). The combinations resulted in 8 treatments: BC0×CK, BC0×OTC, BC0×PE, BC0×PE+OTC, BC1×CK, BC1×OTC, BC1×PE, BC1×PE+OTC.

Each treatment was replicated three times, and a total of 24 pots were used for the experiment. The pots (10 cm top diameter, 6 cm bottom diameter, 8 cm height) had a soil capacity of 1 kg. Each pot was filled with 800 g of soil, amended with 200 g of well-decomposed farmyard manure (FYM), and a commercial NPK fertilizer containing 10% N, 10% P_2_O_5_, and 10% K_2_O.

The concentration of polyethylene (PE = 10 g kg^-1^) was selected to represent a moderate to high level of microplastic contamination commonly reported in agricultural soils receiving repeated plastic mulch film application and sewage sludge amendment (Li et al., 2023). The oxytetracycline concentration (OTC = 50 mg kg^-1^) reflects an environmentally relevant level found in soils following long-term application of livestock manure from treated animals (Khan et al., 2024b). The biochar application rate (20 t ha^-1^) is within the typical recommended range (10–40 t ha^-1^) for soil carbon sequestration and contaminant immobilization in agricultural systems (Khan et al., 2021).

Regarding the potential confounding effects of manure and fertilizer: We acknowledge that the addition of farmyard manure (200 g per pot) and NPK fertilizer was intended to simulate realistic field conditions where organic amendments and synthetic fertilizers are routinely applied to support crop growth. However, we recognize that these inputs may interact with PE and OTC by providing additional organic carbon and nutrients that could influence microbial activity and pollutant bioavailability. To minimize confounding effects, the same manure and fertilizer amounts were applied uniformly across all treatments, allowing direct comparison between contaminated and non-contaminated conditions.

### 2.3 Growth conditions

Following a 2-week stabilization period, five uniform *Amaranthus tricolor* L. seeds were surface-sterilized using 70% ethanol for 30 seconds, rinsed three times with distilled water, and sown at a depth of 1 cm per pot. After germination, seedlings were thinned to one plant per pot. The experiment was conducted under natural greenhouse conditions at the South China Botanical Garden (CAS), Guangzhou, China (23°10’ N, 113°21’ E), with an average temperature of 25 ± 3 °C, relative humidity of 70 ± 5%, and a natural photoperiod of approximately 12–13 hours of daylight; no supplemental lighting or heating was provided. Plants were irrigated daily with tap water to maintain soil moisture at approximately 70% of field capacity, as determined gravimetrically, and no additional fertilization was applied beyond the initial NPK and farmyard manure (FYM) amendment to avoid confounding treatment effects. The 60-day growth period was selected based on the agronomic characteristics of *A. tricolor*, a fast-growing leafy vegetable typically harvested 50–65 days after sowing for optimal tenderness, marketable yield, and edible quality, allowing sufficient time for the establishment of treatment effects (PE, OTC, and biochar) on the soil-plant system while avoiding senescence-related declines in plant physiological activity.

### 2.4 Soil sampling and physicochemical analysis

Soil samples were collected from the 0–10 cm depth using an auger and placed in sealed plastic bags to minimize moisture loss. Sampling was conducted on day 60 after harvest. Soil pH was measured by mixing 5 g of air-dried soil with distilled water (1:5 w/v) in 50 mL centrifuge tubes (Tang et al., 2023). Cation exchange capacity (CEC) was determined by treating 4 g of air-dried soil with ammonium acetate and acetic acid solutions, with two repetitions for each sample. Nitrate (NO_3_^-^-N) and ammonium (NH_4_^+^-N) concentrations were determined from 5 g of fresh soil, analyzed using a UV spectrophotometer at 667 nm and 540 nm, respectively (Khan et al., 2023).

### 2.5 Plant photosynthesis, growth, and antioxidant activities

Leaf gas exchange parameters, including transpiration rate (Tr) (mmol H_2_O m^-2^ s^-1^), net photosynthetic rate (Pn), stomatal conductance (Gs), intercellular CO_2_ concentration (Ci), were measured using a portable photosynthesis system (LI-3200XT, LI-COR Inc., Lincoln, NE, USA). Measurements were recorded between 08:00-11:00 AM on day 60, with the system set at 26 °C, a flow rate of 1000 mL min^-1^, and ambient CO_2_ concentration (400-500 μmol/mol) as followed by the procedure (Sevgi et al., 2026).

After taking photosynthetic measurements, the plants were harvested, and their roots and shoots were rinsed with deionized water and separated. Fresh and dry weights of roots and shoots were recorded. Fresh tissues were used to determine the activities of antioxidant enzymes, including superoxide dismutase (SOD), peroxidase (POD), ascorbate peroxidase (APX), and catalase (CAT), following established protocols (Tang et al., 2023).

### 2.6 Root scanning

After carefully detaching the roots, they were thoroughly cleaned with deionized water to remove soil particles. To determine the root traits (total root area, average root diameter, root volume, surface area, and quantity of root branches), roots were analyzed using WinRhizo TM scanner-based system (v.2007; Reagent Instrument Inc., Quebec City, Canada) (Khan et al., 2024b).

### 2.7 Soil greenhouse gas sampling and analysis

The samples for determining CO_2_ levels were collected between 08:00 and 11:00 AM on days 30 and 60 after sowing, using static closed chambers placed over the soil surface. Gas samples were taken at 0, 10, 20, and 30 minutes using 30 mL plastic syringes and injected into 60 mL gas-tight sampling bags. Samples were analyzed using gas chromatography (Agilent 7890A; Agilent Technologies, Santa Clara, CA, USA) as followed the procedure (Khan et al., 2024b). We acknowledge that CO_2_ emissions were measured at only two time points (days 30 and 60), capturing endpoints rather than temporal dynamics. This sampling schedule was adopted to minimize experimental disturbance, but it precludes assessment of emission patterns across the full growth cycle.

### 2.8 Statistical analysis

All statistical analyses were performed using R software, version 4.1.1 (McMurdie et al., 2013). A two-way analysis of variance (ANOVA) was conducted to evaluate the main and interactive effects of biochar (Factor A) and soil contaminants (Factor B: CK, OTC, PE, PE+OTC) on all measured variables (soil properties, plant growth parameters, antioxidant enzyme activities, and CO_2_ emissions). Following ANOVA, *post hoc* multiple comparisons were performed using Fisher’s Least Significant Difference (LSD) test at a significance threshold of *P < 0.05*. Statistical significance levels are denoted as follows: ****P < 0.001; ***P < 0.01; **P < 0.05; *P < 0.1; and “ns” or no letters indicate non-significant differences (*P ≥ 0.05*). All data are presented as mean ± standard error (SE) of three independent biological replicates (n = 3 per treatment). Variance partitioning analysis was used to assess the correlation between root and leaf antioxidant enzyme activities. Normality of residuals and homogeneity of variances were verified using Shapiro-Wilk and Levene’s tests, respectively, prior to ANOVA.

## 3 Results

### 3.1 Impact of biochar and soil pollutants (PE and OTC) on soil chemical properties

Biochar application enhanced soil fertility, whereas PE and OTC contamination, particularly their combination, degraded soil chemical properties. The incorporation of biochar, soil pollutants (PE and OTC), or their combination significantly influenced the soil chemical properties, including NO_3_^-^-N and NH_4_^+^-N, CEC, and soil pH (*P < 0.01*; **Table 1**). Corn biochar had a more pronounced effect, increasing CEC, NO_3_^-^-N, and NH_4_^+^-N contents by 5%, 22%, and 19%, respectively, compared with the control (*P < 0.01*; **Table 1**). Additionally, biochar application enhanced the soil pH, available nitrogen (AN) by 4%, available phosphorus (AP) by 26%, and available potassium (AK) by 13% compared to the control. Among the various contaminant treatments, the combined application of PE+OTC had the more adverse impact, significantly reducing the soil pH and decreasing AN, AP, AK, CEC, NO_3_^-^-N, and NH_4_^+^-N, contents by 11%, 5%, 13%, 14%, 64%, and 30%, respectively, compared to other treatments (**Table 1**). However, the application of biochar slightly mitigated the adverse effects of PE, OTC, and their combined exposure (PE+OTC) on the soil properties (*P < 0.01*; **Table 1**).

**Table 1:** The impact of biochar and soil pollutants on chemical properties of soil.

Treatment	Soil pH	AN mg kg^- 1^	AP mg kg^- 1^	AK mg kg^- 1^	NH_4_^+^-N (μg/g)	NO_3_^-^-N (μg/g)	CEC (cmol/kg)
B0×CK	6.54 ± 0.01d	67.7 ± 0.7cd	34.7 ± 1.1	107 ± 2.0f	8.58 ± 0.2c	15.3 ± 0.1	21.7 ± 0.2
B0×PE	6.47 ± 0.04e	65.1 ± 1.3e	33.2 ± 1.7	104 ± 0.5g	7.30 ± 0.3e	11.4 ± 0.1	20.4 ± 0.1
B0×OTC	6.38 ± 0.03f	69.9 ± 1.5b	33.9 ± 1.4	114 ± 2.0e	7.80 ± 0.3d	13.1 ± 0.2	19.8 ± 0.6
B0×OTC+PE	6.30 ± 0.02g	62.1 ± 1.7f	31.9 ± 1.6	103 ± 1.6g	6.47 ± 0.2f	9.00 ± 0.3	19.2 ± 0.6
B1×CK	7.42 ± 0.01a	74.2 ± 1.9a	42.0 ± 1.3	148 ± 2.4b	11.0 ± 0.1a	19.0 ± 0.2	22.3 ± 0.8
B1×PE	7.38 ± 0.01a	67.6 ± 0.6cd	38.7 ± 1.1	133 ± 2.2d	8.29 ± 0.2c	15.0 ± 0.4	22.1 ± 1.4
B1×OTC	7.20 ± 0.03b	69.4 ± 1.0bc	40.6 ± 1.8	155 ± 1.8a	9.19 ± 0.3b	16.8 ± 0.4	21.5 ± 0.7
B1×OTC+PE	7.10 ± 0.02c	65.9 ± 0.5de	36.0 ± 1.3	141 ± 2.1c	8.57 ± 0.1c	11.9 ± 0.4	19.5 ± 0.3
B0	6.42 ± 0.11B	66.2 ± 3.3B	33.4 ± 1.2B	107 ± 4.8B	7.54 ± 0.9B	12.2 ± 2.7B	20.3 ± 1.1B
B1	7.27 ± 0.15A	69.3 ± 3.6A	39.3 ± 2.6A	144 ± 9.3A	9.26 ± 1.2A	15.7 ± 3.0A	21.3 ± 1.3A
CK	6.98 ± 0.62A	70.9 ± 4.6A	38.3 ± 5.1A	128 ± 29B	9.78 ± 1.7A	17.2 ± 2.6A	22.0 ± 0.4A
PE	6.92 ± 0.64B	66.4 ± 1.8B	35.9 ± 3.9B	119 ± 20D	7.80 ± 0.7C	13.2 ± 2.6C	21.3 ± 1.2AB
OTC	6.79 ± 0.58C	69.6 ± 0.3A	37.2 ± 4.8AB	134 ± 29A	8.49 ± 1.0B	14.9 ± 2.6B	20.6 ± 1.3B
OTC+PE	6.70 ± 0.57D	64.0 ± 2.7C	34.0 ± 2.9C	122 ± 27C	7.52 ± 1.5D	10.4 ± 2.0D	19.4 ± 0.3C

BC0, no biochar; BC1, corn biochar; CK, Control; PE, Polyethylene; OTC, oxytetracycline; AN, available nitrogen; AP, available phosphorus; AK, available potassium; CEC, cation exchange capacity. Each value shows the mean of three replication ± standard error. Different upper-case letters shown substantial differences (p <0.05) between the main treatments. Lower case letters shown the substantial differences (p <0.05) between interaction of biochar and soil contaminants.

### 3.2 Impact of biochar and soil pollutants (PE and OTC) on plant growth and root development

**Figure 1:**
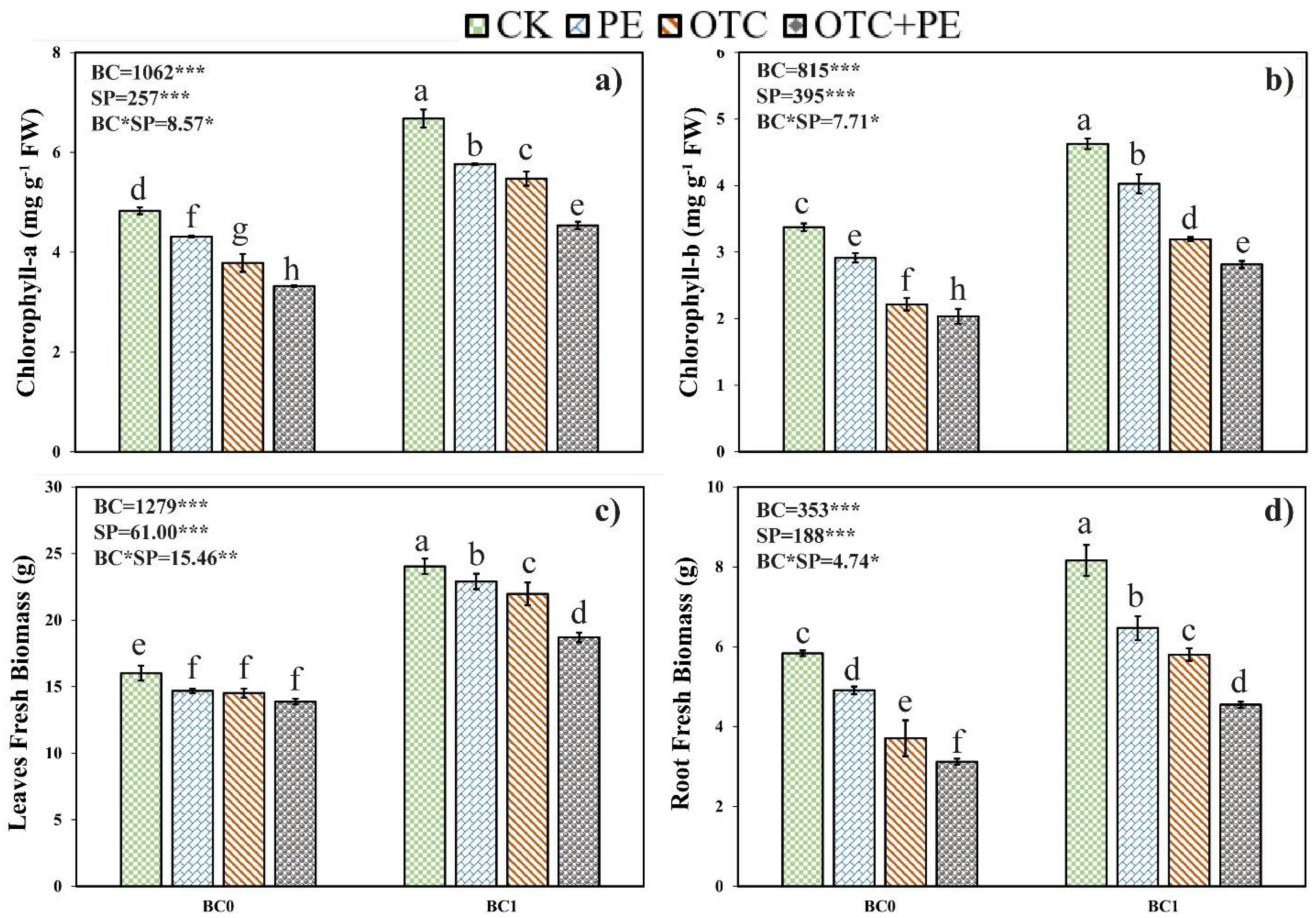
The impacts of biochar and soil pollutants on chlorophyll content and fresh biomass of leaves and roots. BC0, no biochar; BC1, corn biochar; CK, Control; PE, Polyethylene; OTC, oxytetracycline. Vertical error bars represent the standard error (SE) of treatment means (n = 3). LFW, Leaves fresh biomass; RFW, root fresh biomass. Using a two-way ANOVA followed by LSD’s test. ***(P < 0.001); **(P < 0.01); *(P < 0.05).

Biochar promoted plant growth and root development, while PE and OTC contamination suppressed them, with the combined PE+OTC treatment causing the most severe inhibition. The addition of biochar, PE, OTC, and PE+OTC, either alone or in combination, significantly affected the growth, root parameters, and biomass of *A. tricolor* (*P < 0.01*; **Tables 2**, **3**, **Figure 1**). Corn biochar significantly improved both leaf physiological and root morphological parameters compared to the control. OTC and PE (alone and combined) reduced the net photosynthetic rate, transpiration rate, and chlorophyll a and b contents, whereas biochar alleviated these phytotoxic effects (**Table 2**, **Figure 1**).

**Table 2:** The impact of biochar and soil pollutants on plant physiological parameters.

Treatment	Rh (%)	Pn (μmol CO_2_ m^-2^s^-1^)	PAR (μmol CO_2_ m^-2^s^-1^)	CO_2_ (ppm)	Tr H_2_O m^-2^ s^-1^)	Gs (μmol CO_2_ m^-2^s^-1^)	LN
BC0×CK	73.9 ± 1.7b	117 ± 2.7	84.2 ± 2.8e	219 ± 2.0	12.0 ± 0.6b	0.21 ± 0.00c	2.12 ± 0.01
BC0×PE	54.0 ± 2.4ef	114 ± 1.4	78.2 ± 1.8ef	215 ± 2.3	7.89 ± 0.8d	0.18 ± 0.01d	2.10 ± 0.12
BC0×OTC	65.4 ± 2.1d	107 ± 2.0	80.5 ± 2.0fg	210 ± 3.2	7.17 ± 0.2d	0.16 ± 0.01e	2.09 ± 0.03
BC0×OTC+PE	45.6 ± 1.2g	86.7 ± 3.0	74.4 ± 2.5g	202 ± 3.3	7.87 ± 2.0d	0.11 ± 0.01f	2.00 ± 0.02
BC1×CK	85.2 ± 1.4a	162 ± 2.6	122 ± 3.1a	285 ± 2.4	16.1 ± 0.3a	0.29 ± 0.01a	2.97 ± 0.06
BC1×PE	57.0 ± 1.4e	156 ± 2.5	110 ± 2.7b	279 ± 2.6	10.6 ± 0.6bc	0.25 ± 0.01b	2.91 ± 0.08
BC1×OTC	69.1 ± 1.9c	145 ± 2.1	105 ± 1.6c	273 ± 2.1	9.85 ± 0.3c	0.26 ± 0.01b	2.79 ± 0.04
BC1×OTC+PE	51.3 ± 2.2f	130 ± 2.9	99.1 ± 3.2d	265 ± 1.5	7.95 ± 0.2d	0.15 ± 0.01e	2.74 ± 0.03
BC0	59.7 ± 12B	106 ± 14B	79.3 ± 4.1B	212 ± 7.7B	8.73 ± 2.2B	0.17 ± 0.01B	2.08 ± 0.05B
BC1	65.7 ± 15A	148 ± 14A	109 ± 9.5A	276 ± 8.7A	11.1 ± 3.5A	0.24 ± 0.06A	2.85 ± 0.11A
CK	79.6 ± 8.0A	139 ± 32A	103 ± 26A	252 ± 47A	14.0 ± 2.9A	0.25 ± 0.06A	2.54 ± 0.16A
PE	55.5 ± 2.2B	135 ± 30B	94.0 ± 22B	247 ± 45B	9.25 ± 1.9B	0.21 ± 0.05B	2.51 ± 0.57AB
OTC	67.3 ± 2.6C	126 ± 27C	92.8 ± 18B	242 ± 45C	8.51 ± 1.9BC	0.21 ± 0.07B	2.44 ± 0.49BC
OTC+PE	48.5 ± 4.0D	108 ± 30D	86.8 ± 17C	233 ± 45D	7.91 ± 0.1C	0.13 ± 0.03C	2.37 ± 0.52C

BC0, no biochar; BC1, corn biochar; CK, Control; PE, Polyethylene; OTC, oxytetracycline. net photosynthesis (Pn), carbon dioxide (CO_2_), transpiration (Tr), stomatal conductance (Gs), leaf nitrogen (LN), relative humidity (Rh), photosynthetically active radiation (PAR). Vertical error bars represent the standard deviation of treatment means (n = 3). Different lowercase letters indicate significant differences among contaminant treatments (CK, OTC, PE, PE+OTC) within the same biochar level (BC0 or BC1). Different uppercase letters indicate significant differences between biochar levels (BC0 vs. BC1) within the same contaminant treatment.

**Table 3:** The impact of biochar and soil pollutants on plant root parameters.

Treatment	Roots length (cm)	Project area (cm^2^)	Surface area	Average diameter (mm)	Root tips
BC0×CK	211 ± 6.1b	11.6 ± 0.6b	16.2 ± 0.7b	0.82 ± 0.03	2496 ± 143d
BC0×PE	245 ± 20b	8.36 ± 0.2cd	12.6 ± 0.4d	0.57 ± 0.02	2519 ± 293d
BC0×OTC	238 ± 11b	8.08 ± 0.2cd	12.2 ± 0.3d	0.44 ± 0.02	2254 ± 117ef
BC0×OTC+PE	221 ± 29b	6.73 ± 0.4e	9.40 ± 0.4f	0.33 ± 0.03	2120 ± 95.5f
BC1×CK	318 ± 10a	14.5 ± 0.3a	18.9 ± 0.2a	0.98 ± 0.10	3582 ± 42.2a
BC1×PE	248 ± 31b	8.67 ± 0.3c	14.9 ± 0.3c	0.69 ± 0.03	3180 ± 34.7b
BC1×OTC	237 ± 21b	8.25 ± 0.2d	12.8 ± 0.2d	0.53 ± 0.02	2779 ± 75.5c
BC1×OTC+PE	220 ± 38b	6.52 ± 0.2e	11.0 ± 0.3e	0.45 ± 0.01	2435 ± 55.3de
BC0	229 ± 15B	8.68 ± 2.0B	12.6 ± 2.8B	0.54 ± 0.21B	2347 ± 193B
BC1	256 ± 43A	9.48 ± 3.5A	14.4 ± 3.4A	0.66 ± 0.23A	2994 ± 496A
CK	265 ± 75A	13.0 ± 2.1A	17.5 ± 1.9A	0.90 ± 0.11A	3039 ± 768A
PE	246 ± 2.1AB	8.51 ± 0.2B	13.8 ± 1.6B	0.63 ± 0.08B	2849 ± 468B
OTC	238 ± 0.4AB	8.16 ± 0.1B	12.5 ± 0.4C	0.49 ± 0.06C	2516 ± 371C
OTC+PE	221 ± 0.7B	6.63 ± 0.1C	10.2 ± 1.1D	0.39 ± 0.09D	2278 ± 223D

BC0, no biochar; BC1, corn biochar; CK, Control; PE, Polyethylene; OTC, oxytetracycline. Each value shows the mean of three replication ± standard error. Different upper-case letters shown substantial differences (p <0.05) between the main treatments, while the lower-case letters shown the substantial differences (p <0.05) between interaction of biochar and soil pollutants.

Biochar application substantially improved root length, project area, surface area, average diameter, and root tips in soil contaminated with PE and OTC. Conversely, the sole or combined application of PE and OTC significantly reduced root parameters (*P < 0.01*; **Table 3**). The combined exposure (OTC + PE) had the most detrimental effect on root growth (*P < 0.01*; **Table 3**). In contrast, biochar application (20 t ha^-1^) significantly enhanced the root growth parameters compared with the control (0 t ha^-1^).

The addition of corn biochar increased leaf and root biomass by 33% and 30%, respectively, compared to the no-biochar treatment (**Figure 1**). Conversely, soil contaminated with OTC+PE had more pronounced negative impacts, reducing leaf and root biomass by 23% and 45%, respectively, compared to soils with single pollutants (PE and OTC). Biochar addition to contaminated soil (PE and OTC) resulted in a slight increase in fresh biomass of *A. tricolor*, with better performance observed under single-pollutant than combined-pollutant treatments (**Figure 1**).

### 3.3 Impact of biochar and soil pollutants (PE and OTC) on leaf and root antioxidant activities

**Figure 2:**
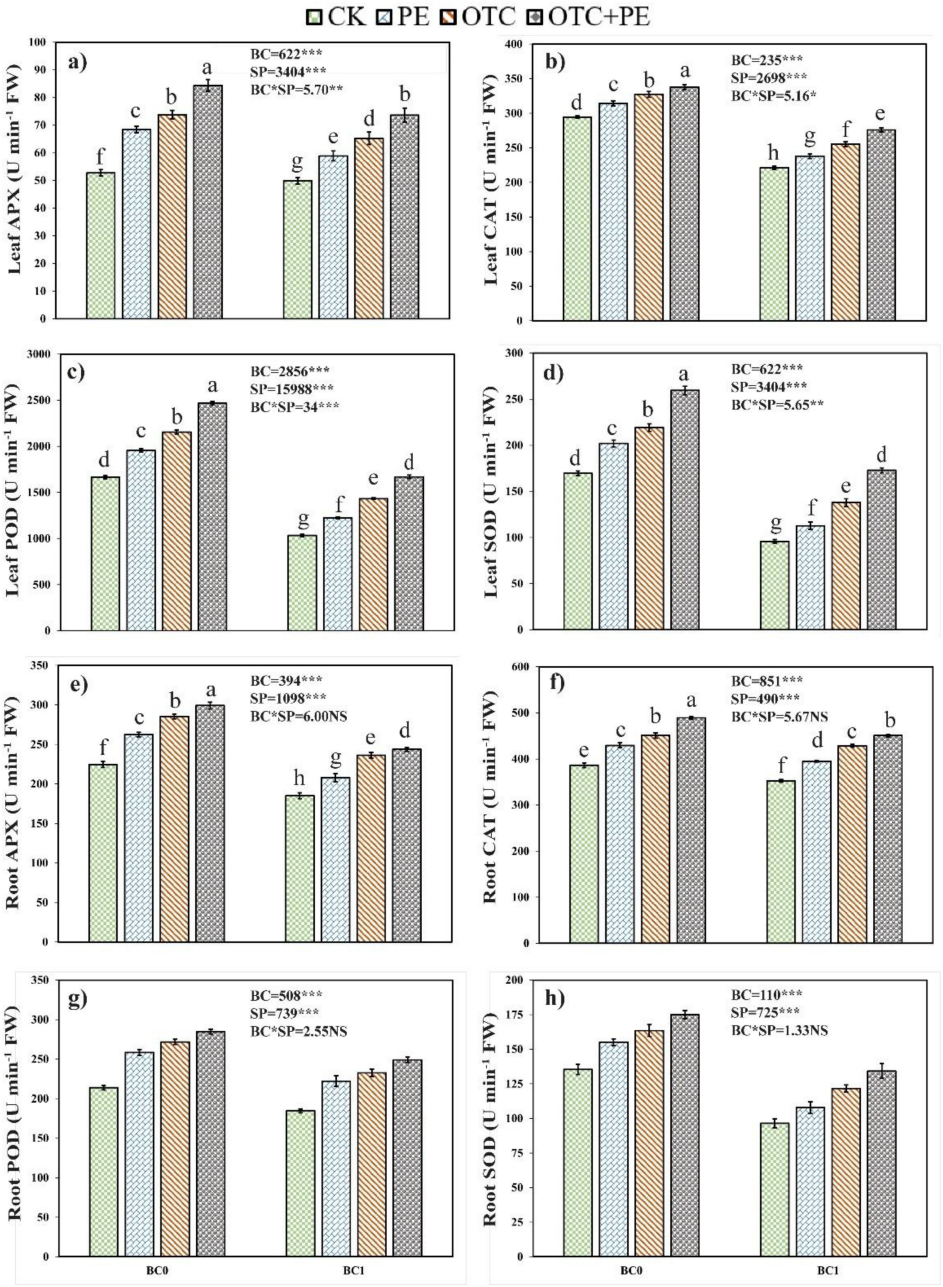
**(a-d)** show the leaf antioxidants enzyme activities under different treatments of soil. **(e-h)** Root antioxidant enzyme activities under different treatments of soil. BC0, no biochar; BC1, corn biochar; CK, Control; PE, Polyethylene; OTC, oxytetracycline. APX, ascorbate peroxidase; CAT, catalase; POD, peroxidase; SOD, superoxide dismutase. Vertical error bars represent the standard error of treatment means (n = 3). Using a two-way ANOVA followed by LSD’s test. Using a two-way ANOVA followed by LSD’s test. ***(P < 0.001); **(P < 0.01); *(P < 0.05). no letters = showed non-considerable differences.

Biochar suppressed antioxidant enzyme activities by reducing oxidative stress, while PE and OTC contamination, especially their combination, stimulated antioxidant activity as a stress response. The application of biochar, PE, OTC, and PE+OTC, either individually or in combination, significantly influenced enzymatic antioxidants activities (APX, CAT, POD, and SOD) in both leaves and roots (**Figure 2**). Solely application of biochar suppressed leaves antioxidant enzyme activities up to 11-39 (APX:11%, CAT:22%, POD:35%, and SOD:39%), while in roots up to 7-27% (APX:19%, CAT:7%, POD:14%, and SOD:27%), respectively. Contrarily, soil pollutants application PE, OTC, and their combined exposure (PE+OTC) stimulated antioxidant activity, particularly under combined exposure, which led to higher toxicity, increasing antioxidant activity in leaves by 39%, 35%, 16%, and 35%, and in roots by 25%, 21%, 25%, and 25%, respectively, compared to single pollutants application (**Figure 2**). Corn biochar mitigated the stimulatory effect of soil pollutants (PE and OTC) on both leaves and root antioxidants. In PE and OTC-contaminated soils, the BC0 treatment significantly increased antioxidant levels, while BC1 significantly reduced them compared to other treatments. Furthermore, PE, OTC, and PE+OTC notably inhibited APX activity, with the combined treatment (PE+OTC) exhibiting more substantial toxicity than either individual contaminant (**Figure 2**).

### 3.4 Impact of biochar and soil pollutants (PE and OTC) on soil CO_2_ emissions

**Figure 3:**
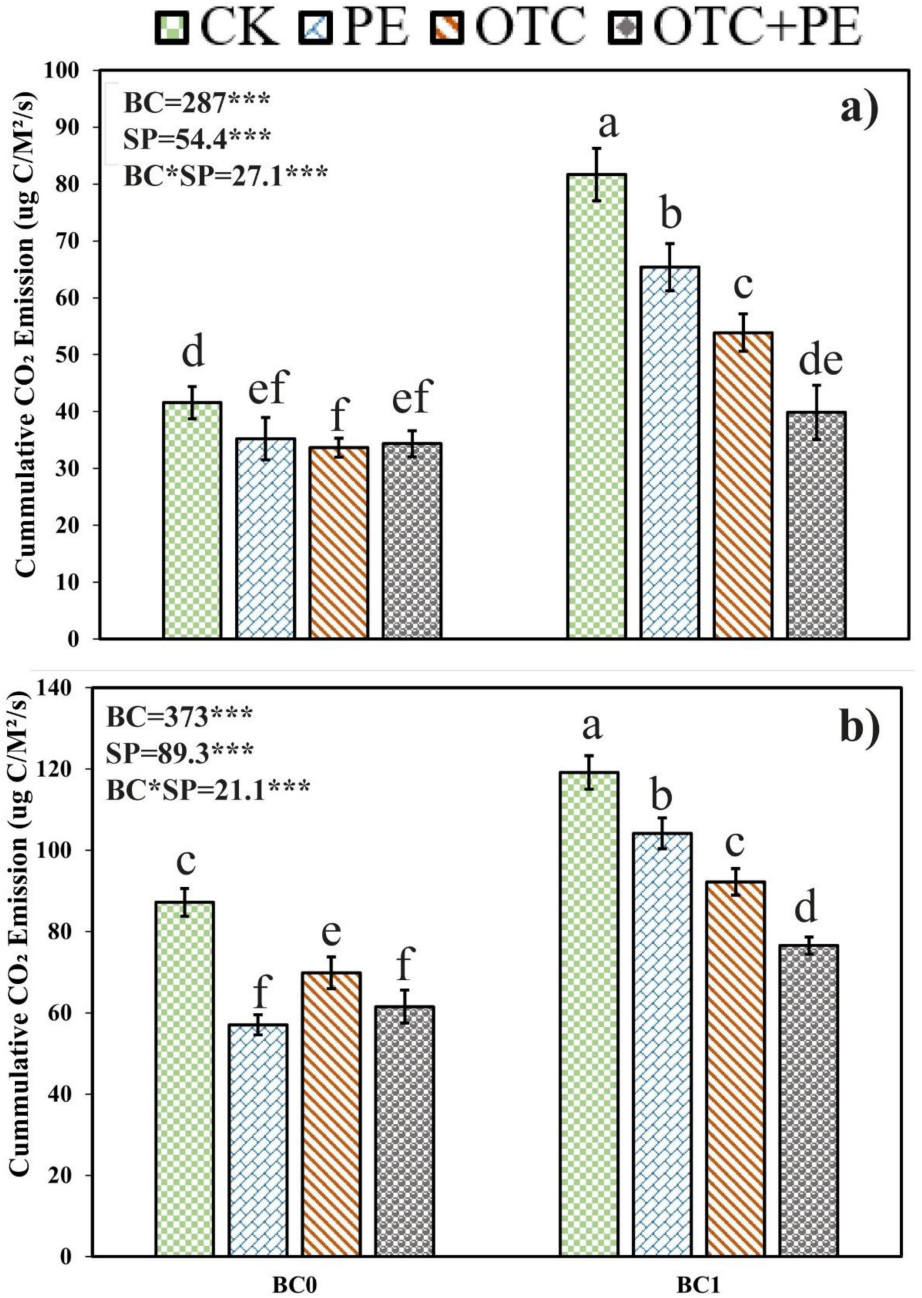
The impact of biochar and soil pollutants on CO_2_ gas emission. **(a)** represents CO_2_ emissions at 30 days; **(b)** represents CO_2_ emissions at 60 days. BC0, no biochar; BC1, corn biochar; CK, control; PE, polyethylene; OTC, oxytetracycline. Vertical error bars represent the standard error of treatment means (n = 3). Statistical analysis was performed using two-way ANOVA followed by LSD’s test. Significance levels: Using a two-way ANOVA followed by LSD’s test. ***(P < 0.001); **(P < 0.01); *(P < 0.05).

The individual and combined application of biochar, PE, and OTC significantly reduced CO_2_ emissions at both 30 and 60 days after sowing. Corn biochar alone increased CO_2_ emissions by 39% and 30% at 30 and 60 days, respectively, compared with the control (*P < 0.01*; **Figure 3**). Among all treatments, PE+OTC resulted in the most significant decline in CO_2_ emissions, reducing them by 55% and 28%, respectively, compared to other treatments (**Figure 3**). The highest CO_2_ emissions (49% and 33%) were recorded in the BCI × CK treatment, followed by BC1 × PE and other combinations (*P < 0.01*; **Figure 3**). Overall, biochar increased CO_2_ emissions by enhancing microbial activity, while PE and OTC contamination, particularly their combination, significantly reduced CO_2_ emissions.

### 3.5 Correlation between leaf and root parameters and soil properties

**Figure 4:**
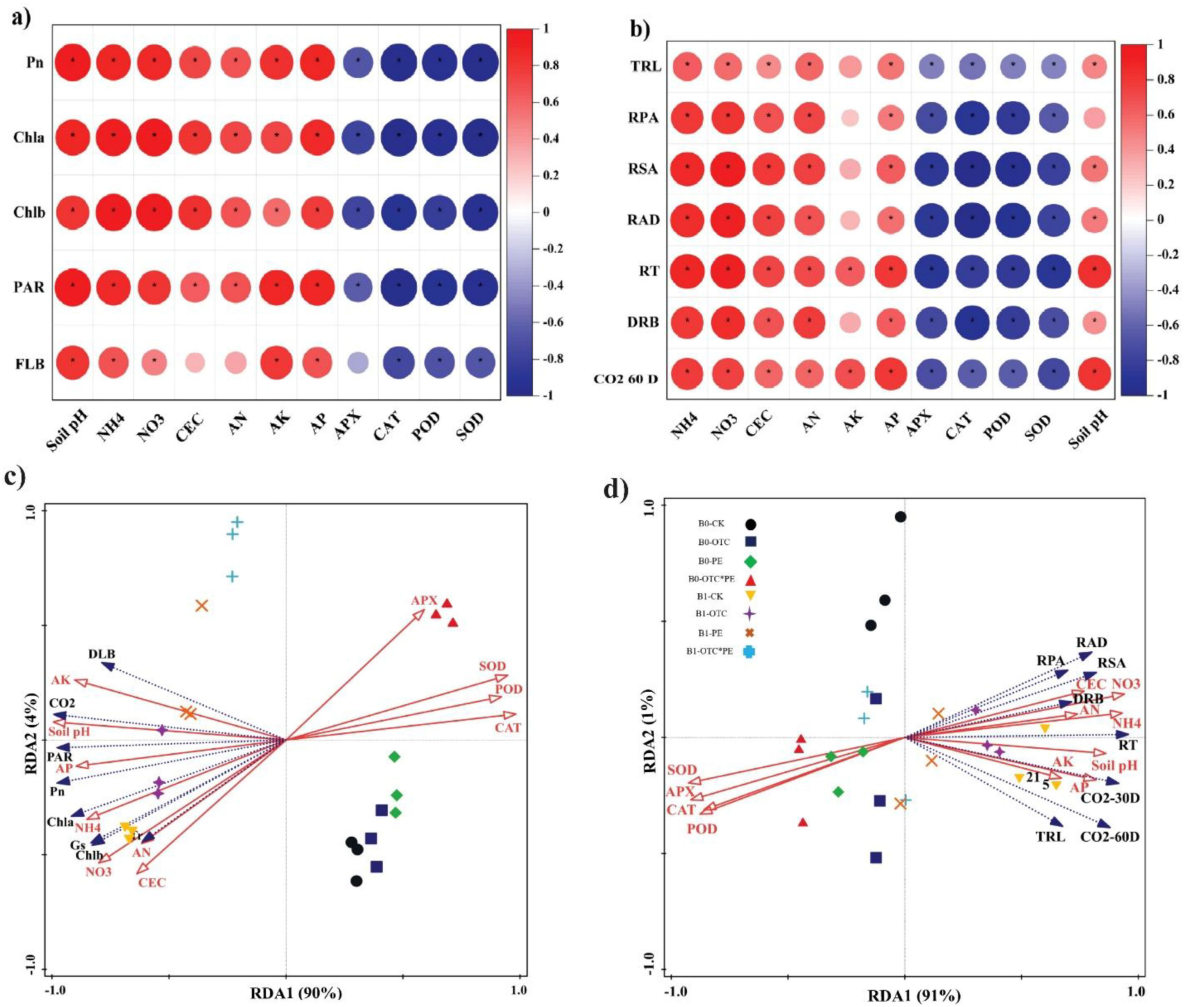
**(a)** shows the correlation between the photosynthetic, chlorophyll contents and leaves biomass with leaves antioxidants. The correlation between the root traits with root antioxidants is shown in **(b)**. FLB= fresh leaves biomass, DRB= root dry biomass root total length, RSA= root surface area, RPA= root project area, RAD= root average diameters, RT= root tips. **(c)** Redundancy analysis based on *Amaranth tricolor* leaves physiological parameters and soil parameters under solely and co-exposure treatment with biochar, polyethylene (PE), and oxytetracycline (OTC). **(d)** Redundancy analysis based on *Amaranth tricolor* root traits and soil parameters under solely and co-exposure treatment with biochar, polyethylene (PE), and oxytetracycline (OTC). * (P < 0.05).

Strong positive correlations were observed between soil properties and plant growth parameters, while negative correlations existed between antioxidant activities and plant biomass, indicating stress-induced oxidative damage. Correlation analysis revealed that photosynthetic parameters, chlorophyll content, and leaf biomass were significantly correlated with soil properties and leaf antioxidant activities (**Figures 4a, b**). Leaf biomass, photosynthesis, and chlorophyll content showed significant negative correlations with leaf antioxidants (APX, POD, SOD, and CAT) (**Figure 4a**; *P< 0.05*). In contrast, soil physicochemical properties were strongly and positively correlated with photosynthesis, chlorophyll content, and leaf biomass (**Figure 4a**). Moreover, CO_2_ emissions, root traits, and biomass were significantly associated with root antioxidants and soil physicochemical properties (**Figure 4b**; *P < 0.05*). A strong negative correlation was observed between root traits (root length, surface area, projected area, average diameters, and tips) and root biomass, as well as root antioxidant activities (**Figure 4b**, *P < 0.05*). A strong positive correlation existed between these root traits and soil physiochemical properties (**Figure 4b**). The Redundancy Analysis (RDA) demonstrated treatment-induced variability in leaf and root parameters, as RDA1 explained 92% (leaf) and 91% (root) of the variance, and RDA2 explained 4% and 1%, respectively (**Figures 4c, d**). Collectively, these soil parameters accounted for the observed variation in both root and leaf characteristics.

### 3.6 Partial least squares path modeling of soil-plant relationships

**Figure 5:**
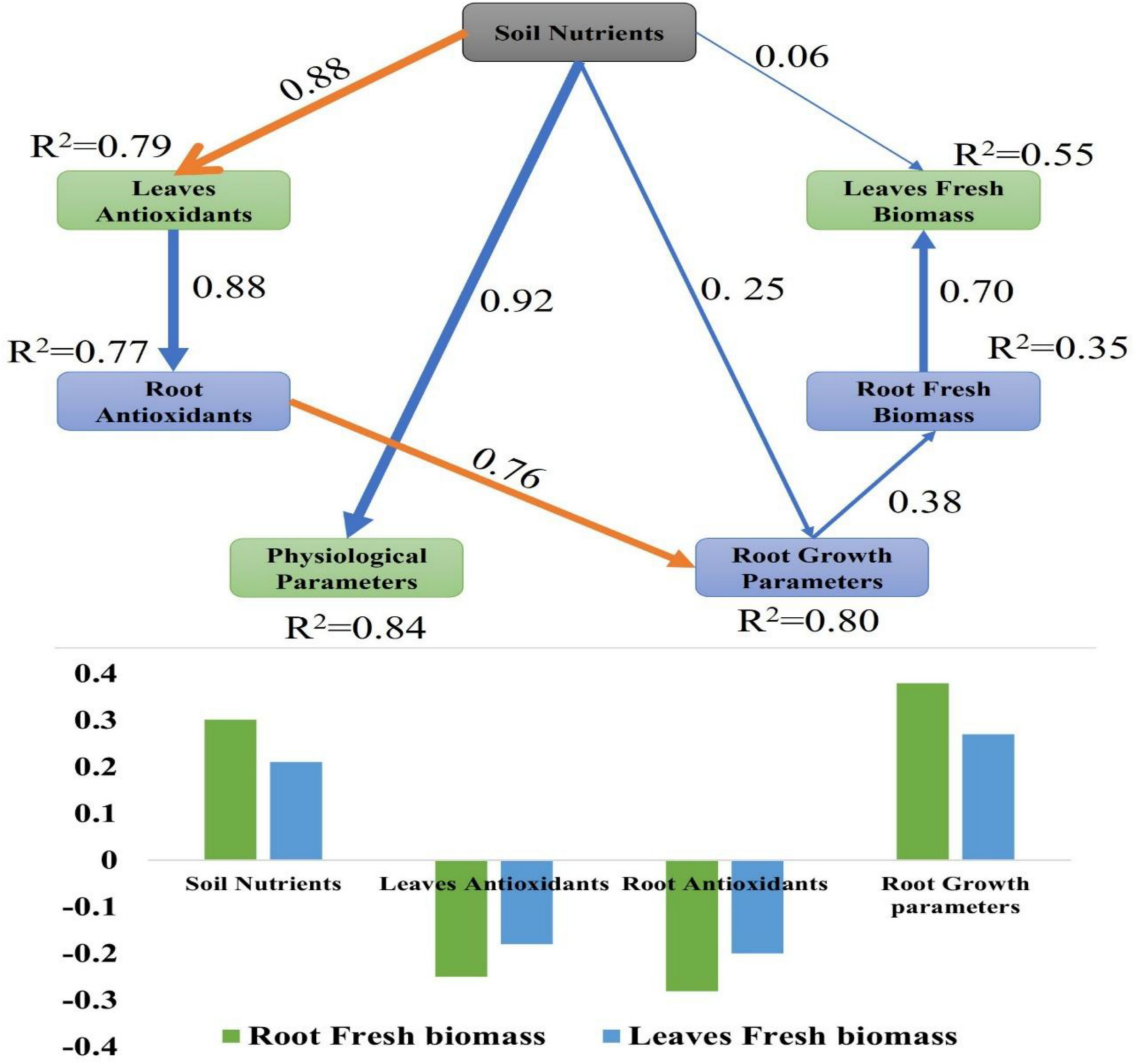
Partial least squares path model showing direct and indirect relationships among soil nutrients, antioxidant responses, physiological traits, root growth, and biomass of *Amaranthus tricolor* under biochar, polyethylene, and oxytetracycline treatments. Blue arrows indicate positive pathways, orange arrows indicate negative or stress-associated pathways, and numbers on arrows represent standardized path coefficients. R^2^ values indicate the proportion of variance explained for each endogenous variable. The lower bar plot shows the relative direct effects of soil nutrients, leaf antioxidants, root antioxidants, and root growth parameters on root and leaf fresh biomass.

The partial least squares path model showed that soil nutrients were the central driver of the soil–plant response network, explaining 84% of the variation in physiological parameters and strongly influencing root growth parameters. Soil nutrients had a strong direct positive effect on physiological parameters (path coefficient = 0.92) and a weaker direct effect on root growth parameters (0.25), whereas their direct effect on leaf fresh biomass was minimal (0.06). Leaf and root antioxidant responses were strongly associated, as leaf antioxidants positively influenced root antioxidants (0.88). Root antioxidants showed a strong positive association with root growth parameters (0.76), suggesting that antioxidant activation was closely linked to root stress adjustment. Root growth parameters positively affected root fresh biomass (0.38), and root fresh biomass subsequently contributed strongly to leaf fresh biomass (0.70). The model explained substantial variation in leaf antioxidants, root antioxidants, physiological traits, root growth traits, root biomass, and leaf biomass, with R² values of 0.79, 0.77, 0.84, 0.80, 0.35, and 0.55, respectively (**Figure 5**).

## 4 Discussion

### 4.1 Impact on soil chemical properties

Biochar alone increased CEC (5%), NO_3_^-^-N (22%), NH_4_^+^-N (19%), AN (4%), AP (26%), and AK (13%), while PE+OTC reduced these properties by 11-64% (**Table 1**). Biochar partially mitigated the adverse effects of PE and OTC. The inhibitory effects of PE and OTC on soil properties are likely due to their non-degradable nature and interactions that alter microbial activity and nutrient dynamics (Afzal et al., 2025a). Mechanistically, PE particles adsorb soil nutrients and organic matter, reducing their bioavailability to microbes (Wang et al., 2024a), while OTC disrupts microbial community structure and function (Guo et al., 2022; Meng et al., 2026). The combined PE+OTC treatment exacerbated these effects, likely through co-adsorption processes where PE serves as a vector for OTC (Mejías et al., 2026), thereby increasing localized antibiotic concentrations and prolonging its persistence in soil. Consistent with previous reports, MPs of different sizes and shapes influence soil functions and microbial activity depending on their abundance and exposure duration (Lozano et al., 2021). Biochar alleviated these adverse effects through multiple mechanisms: its high surface area (10.45 m^2^ g^-1^), porous structure (pore diameter 1.93 nm), and oxygen-containing functional groups (FTIR, **Supplementary Figure 1A**) facilitated adsorption and complexation of OTC and PE particles; increased soil CEC (by 5%) enhanced retention of NH_4_^+^, K^+^, and Ca^2+^; and improved soil pH created favorable conditions for nitrifying bacteria and accelerated nitrogen cycling (Khan et al., 2021). These findings align with studied showing that biochar improves microbial richness and functional gene abundance in MPs-contaminated soils (Ran et al., 2023) and with a study reporting that biochar-amended improved soil enzyme activity, bacterial diversity and the soil health, while mitigated the adverse effect of soil contaminants (Khalid et al., 2023).

### 4.2 Impact on plant growth, root parameters, and antioxidant activity

Biochar significantly enhanced plant performance, increasing leaf and root biomass by 33% and 30%, respectively, whereas combined PE+OTC exposure caused substantial reductions up to 23-45% (**Figures 1a–c**). These growth responses were accompanied by marked physiological impairment, as PE, OTC, and their combined treatment reduced net photosynthetic rate, transpiration, and chlorophyll content (**Table 2**), indicating stress-induced disruption of photosynthetic activities. Such reductions are consistent with previous observations that both MPs and ATs can interfere with plant physiological functioning and nutrient dynamics (Bao et al., 2022; Guo et al., 2022). In contrast, biochar partially alleviated these reductions, likely by improving soil conditions and nutrient availability, as reflected in enhanced soil chemical properties (Khalid et al., 2023). Notably, under no-biochar conditions, PE and OTC individually increased leaf fresh biomass slightly compared to control treatment (**Figure 1c**). This unexpected stimulatory effect may be explained by a hormetic response, where low-dose contaminant exposure triggers compensatory growth mechanisms, including enhanced root exudation and nutrient solubilization (Guo et al., 2026). However, this effect was not sustained under combined PE+OTC exposure, where biomass declined significantly.

Root morphological parameters were adversely affected by both individual and combined exposures to PE and OTC. Sole application of these contaminants, as well as their co-exposure, significantly reduced root length, surface area, and branch number (**Table 3**), indicating impaired soil exploration and nutrient acquisition. PE particles (100–200 µm) are too large to penetrate root cells but accumulate in the intercellular layer, physically blocking nutrient transport (Zhou et al., 2021) (Zhou et al., 2021). In contrast, OTC may enter root tissues and interfere with cellular metabolism, thereby limiting energy availability for root growth (Bao et al., 2022). Biochar improved root architecture under both control and contaminated conditions, likely through enhanced nutrient retention and improved soil structure (Yu et al., 2024).

Antioxidant activities (APX, CAT, POD, and SOD) exhibited contrasting patterns across treatments. PE, OTC, and particularly their combined exposure significantly increased antioxidant activities in both leaves and roots (**Figures 2a–h**), reflecting elevated oxidative stress and increased reactive oxygen species (ROS) production. Plants respond to such stress by activating antioxidant defense systems to mitigate cellular damage (Fathi et al., 2025). In contrast, biochar reduced antioxidant enzyme activities (11-39% in leaves; 7-27% in roots), indicating alleviation of oxidative stress, likely due to improved soil conditions and reduced pollutant pressure. Notably, root antioxidant responses were generally stronger than those in leaves, suggesting greater root sensitivity to soil-borne contaminants (Zeng et al., 2025).

The partial least squares path model (**Figure 5**) provides further insight into the relationships among soil properties, plant physiological traits, and biomass accumulation. Soil nutrient status exerted a strong direct influence on physiological parameters, which, in turn, indirectly supported biomass formation. In contrast, the direct effect of soil nutrients on leaf biomass was relatively weak, suggesting that plant growth responses were primarily mediated by physiological performance and root development rather than by direct nutrient effects. The strong association between leaf and root antioxidant responses reflects coordinated systemic stress signaling, while the positive linkage between root antioxidants and root growth parameters suggests that antioxidant activation contributes to maintaining root function under stress conditions. Importantly, root biomass contributed strongly to leaf biomass, highlighting that recovery of root growth is a key pathway through which biochar mitigates PE-OTC-induced phytotoxicity.

### 4.3 Impact on soil CO_2_ emissions

Biochar alone increased CO_2_ emissions by 39% (day 30) and 30% (day 60), while PE+OTC reduced emissions by 55% and 28%, respectively (**Figures 3a, b**). Biochar partially restored CO_2_ emissions in contaminated soils. Soil CO_2_ emissions originate from three primary sources: microbial decomposition of soil organic matter, root autotrophic respiration, and biochar-derived pyrogenic carbon mineralization. The inhibitory effects of PE on CO_2_ emissions are attributed to PE particles adsorbing soil nutrients and organic substrates, reducing their availability to microbes and lowering microbial carbon use efficiency (CUE) (Chang et al., 2024; Khan et al., 2024a). PE also alters soil pH and CEC, creating unfavorable conditions for microbial growth (Chen et al., 2024). OTC adversely affects root growth parameters (length, surface area, branch number), directly reducing root-derived CO_2_ emissions (Khan et al., 2024b). The combined PE+OTC treatment resulted in lower CO_2_ emissions than individual treatments (**Figure 2**), suggesting an antagonistic interaction where PE-mediated nutrient adsorption and OTC-induced root inhibition synergistically limit substrates for microbial respiration (Chen et al., 2023).

Biochar increased CO_2_ emissions through multiple mechanisms: release of labile soluble organic carbon (DOC) that primes microbial activity; improved soil aeration and water holding capacity enhancing microbial habitat; and increased root biomass providing more rhizo-deposits (Li et al., 2022; Chen et al., 2025). The nutrient composition and porosity of biochar (**Supplementary Table 2**) play important roles in modulating these effects (Ji et al., 2025). Notably, co-application of biochar and MPs mitigated global warming potential of cumulative GHG emissions, decreasing CO_2_, N_2_O, and CH_4_ emissions (Khan et al., 2024b).

We acknowledge several limitations: CO_2_ emissions were measured at only two time points (days 30 and 60); the short-term (60 days) greenhouse conditions may not reflect field realities; we did not measure microbial community composition, ARGs, OTC bioavailability, PE-OTC co-sorption, oxidative damage indicators (MDA, H_2_O_2_, O_2_^-^), OTC accumulation in plant tissues, or plant uptake of PE/OTC. Accordingly, the proposed mechanistic framework is presented as a working hypothesis requiring direct validation. Future research should focus on long-term field trials, higher-frequency CO_2_ measurements, quantification of OTC translocation (HPLC-MS/MS), oxidative damage markers, metagenomic analysis of microbial communities and ARGs, and systematic evaluation of biochar feedstock and pyrolysis conditions.

## 5 Conclusion

This study demonstrates that solely application or co-exposure to PE and OTC in soil synergistically inhibits red amaranth growth, photosynthesis, and antioxidant activity. At the same time, biochar amendment effectively mitigates these phytotoxic effects by improving soil properties and reducing oxidative stress. Consistent with our CO_2_ measurements, corn biochar alone stimulated soil respiration and increased CO_2_ emissions, whereas PE and OTC especially in combination significantly suppressed CO_2_ release; when applied together with pollutants, biochar partly offset this inhibition, leading to an overall moderating rather than uniformly reducing effect on soil CO_2_ flux. These findings support the potential of biochar as a remediation strategy for soils co-contaminated with MPs and antibiotics, while highlighting the need to balance its agronomic and remediation benefits with its influence on soil carbon dynamics. However, further research is needed to elucidate the long-term field-scale mechanisms, including impacts on microbial communities and antibiotic resistance genes (ARGs), and to develop practical guidelines for biochar use under complex co-contamination scenarios.

### 5.1 Environmental implication

The widespread prevalence of plastic waste and antibiotics has led to a significant and ongoing accumulation of PE and OTC, particularly in agricultural soil. Exposure to soil contaminants, either alone or in combination, has been shown to have toxic effects on soil nutrients and plant growth. In contrast, biochar positively influences soil nutrients and plant growth while mitigating the adverse effects of both soil contaminants (PE and OTC) and CO_2_ emissions. This study elucidates the adverse impact of PE and OTC on soil physiochemical properties and plant growth. Biochar application is a sustainable technique in farmland that mitigates these effects, addressing the existing knowledge gap regarding their influence on soil health and agricultural cropping systems.

## Data Availability

The original contributions presented in the study are included in the article/**Supplementary Material**. Further inquiries can be directed to the corresponding authors.
