# Supplementary material for: Biochar mitigates polyethylene and oxytetracycline stress in Amaranthus tricolor by improving soil properties, reducing oxidative damage, and moderating CO2 emissions
Source: Front Plant Sci. 2026 Jun 29;17:1829340. doi: 10.3389/fpls.2026.1829340 (PMC13357139; doi:10.3389/fpls.2026.1829340)
Supplement: Supplementary file 1 [file SupplementaryFile1.docx]

**Text S1. Biochar and soil properties:**

The H/C ratio is an indicator of aromaticity; the lower the H/C ratio, the higher the aromaticity. Corn biochar has a higher C/H ratio, indicating a higher degree of carbonization in corn biochar, with more complete π-conjugated structures. The π-π electron donor-acceptor interaction between the π bonds provided by antibiotics and the aromatic rings of biochar is completed through adsorption. High lignin content, resulting in high aromatization after carbonization, contains numerous π-electron acceptors that can form π-π bonds with π-electron donors, such as oxytetracycline (OTC), thereby adsorbing onto the biochar. Corn biochar has a larger specific surface area, providing more adsorption sites for π-π bonding with OTC, thereby enhancing the adsorption capacity of the biochar (Table S2). It is believed that microplastics exhibit strong hydrophobicity, which weakens the polarity during biomass pyrolysis at high temperatures, continuously increasing hydrophobicity, which is also an essential reason for hydrophobic adsorption. Corn biochar has a higher adsorption capacity for PE and OTC.

**Text S2. Characterization of microplastics and biochar**

The surface morphology and elemental composition of PE microplastics were analyzed using Scanning Electron Microscopy (SEM) in conjunction with Energy-Dispersive X-ray Spectroscopy (EDS), with the results illustrated in Figure S1. As shown in Figure S1B, the surface of biochar exhibits a porous and irregular texture, characterized by numerous pores of varying sizes. These pores enhance the contact area between biochar and its surrounding environment, thereby improving its adsorption performance and other functional properties (Ahmed et al., 2016). SEM imaging revealed a highly irregular and textured surface with visible folds, grooves, and fractured regions. These morphological features are characteristic of environmental degradation processes such as mechanical abrasion and photothermal weathering, which are commonly experienced by plastic debris in aquatic and terrestrial ecosystems (Figure S2D).

Fourier Transform Infrared Spectroscopy (FTIR) was employed to analyze the chemical bonds and functional groups present in biochar and PE. As illustrated in Figure S1A, biochar exhibits absorption peaks at various wavenumbers, which correspond to distinct chemical bonds or functional groups. Notably, the strong absorption peak observed at the range of 3433 cm^-1^ suggests the presence of -OH groups, potentially attributable to water molecules or other oxygen-containing functional groups (Liu and Zhang, 2022). The presence of -CH_2_-, C-C, C=O, C-O-C, C=C, and numerous single-bond stretches was also observed on different absorption peaks. PE also presents several prominent absorption peaks that correspond to specific vibrational modes of chemical bonds, including a peak near 3433 cm^-1^ associated with -OH stretching vibrations and a broad peak in the vicinity of 2852-2922 cm^-1^ associated with CH stretching vibrations. The X-ray diffraction (XRD) pattern serves as a crucial indicator for characterizing the crystallinity of microplastic samples, as polymers with high crystallinity typically exhibit distinct diffraction peaks (Shaheen et al., 2022;Sun et al., 2022). As depicted in Figure S1B, the XRD curve for biochar is relatively smooth, lacking significant peaks, which suggests that biochar may be amorphous or possess low crystallinity. In contrast, the PE curve revealed several primary peaks. These XRD results indicate that PE is a partially crystalline polymer.


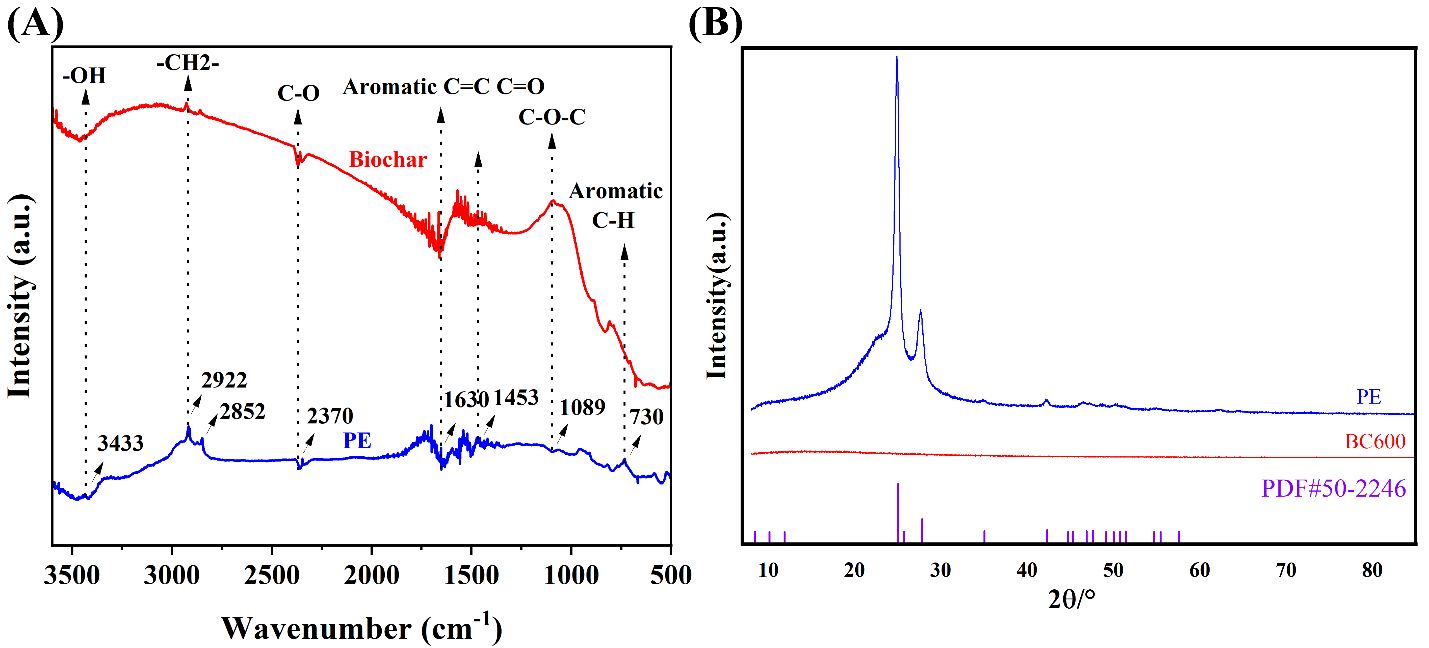
**Figure. S1** FTIR spectra of Biochar and PE (A), the XRD patterns of Biochar and PE (B)


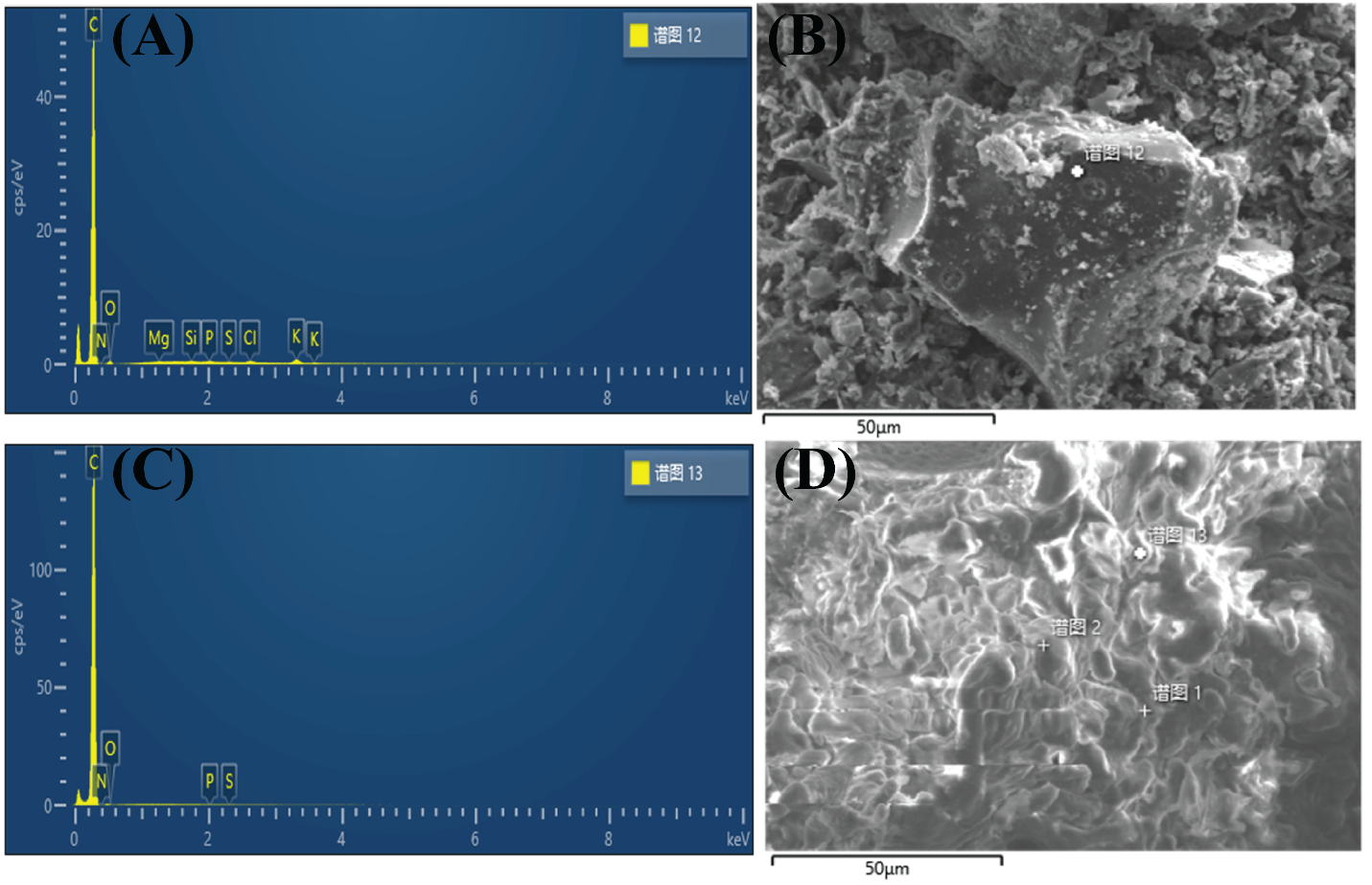


**Figure. S2** SEM and EDS images of biochar (A, C) and PE (C, D).

**Table S1 indicates the soil's physiochemical properties before the experiment.**

| Soil physiochemical properties | Value |
| --- | --- |
| Soil pH | 7.83 ± 0.03 |
| CEC (cmol/kg) | 12.95 ± 0.46 |
| NO_3_^-^-N content (mg kg^-1^) | 15.37 ± 0.35 |
| NH_4_^+^-N content (mg kg^-1^) | 9.86 ± 0.21 |

Note: CEC= Cation exchange capacity

**Table S2 The physiochemical properties of Corn biochar**

|  | BET | Pore Diameter | Pore Volume | Element content (%) | | | Element ratios | |
| --- | --- | --- | --- | --- | --- | --- | --- | --- |
|  | (m^2^ g^-1^) | nm | cm^3^ g^-1^ | N | C | H | C/ N | C/H |
| Corn | 10.45 | 1.93 | 0.005 | 2.71±0.03 | 61.21±1.36 | 1.84±0.03 | 22.74 | 33.26 |

Note: N= nitrogen, C= carbon comtent, H= hydrogen, C/N= carbon and nitrogen ratio, C/H carbon hydrogen ratio

**Table S3. Analysis of variance (ANOVA) for plant physiochemical parameters**

| **Treatment** | **Soil pH** | **CEC** | **AN** | **AP** | **AK** | **NH_4_^+^-N** | **NO_3_^-^-N** |
| --- | --- | --- | --- | --- | --- | --- | --- |
| Biochar | 7169*** | 14.30** | 37.25*** | 100.2*** | 2307*** | 470.8*** | 854.7*** |
| Pollutants | 162.3*** | 15.55*** | 38.39*** | 10.13*** | 77.66*** | 161.7*** | 575.6*** |
| Biochar × pollutants | 6.68** | 1.85ns | 8.090** | 1.340ns | 13.41*** | 16.39*** | 3.060ns |

Note: CEC: cation exchange capacity, AN: available nitrogen, AP: available phosphorus, AK: available potassium

| **Treatment** | **Pn** | **CO_2_** | **Tr** | **Gs** | **LN** | **Rh** | **PAR** | **Chl-a** | **Chl-b** |
| --- | --- | --- | --- | --- | --- | --- | --- | --- | --- |
| Biochar | 1755*** | 4014*** | 47.5*** | 620*** | 1014*** | 63.1*** | 827*** | 1206*** | 858*** |
| Pollutants | 185*** | 66.1*** | 64.5*** | 291*** | 9.98*** | 337*** | 41.7*** | 293*** | 416*** |
| Biochar × pollutants | 2.37ns | 0.42ns | 5.83** | 14.4*** | 14.4*** | 6.40** | 8.83** | 9.73*** | 8.11** |

**Table S4. Analysis of variance (ANOVA) for plant physiological parameters**

Noted: net photosynthesis (Pn), carbon dioxide (CO_2_), transpiration (Tr), stomatal conductance (Gs), leaf number (LN), relative humidity (Rh), photosynthetically active radiation (PAR), chlorophyll a (Chl-a), and chlorophyll b (Chl-b).

| **Treatment** | **Roots length** | **Project Area** | **Surface Area** | **Average diameter** | **Root tips** |
| --- | --- | --- | --- | --- | --- |
| Biochar | 7.83* | 36.0*** | 136*** | 52.4*** | 142*** |
| Pollutants | 3.62* | 418*** | 398*** | 172*** | 39.2*** |
| Biochar × pollutants | 7.59** | 28.5*** | 8.92** | 0.73ns | 9.00** |

**Table S5. Analysis of variance (ANOVA) for root physiological parameters**

**Table S6. Analysis of variance (ANOVA) for leaves and root antioxidants**

|  | **Leaves antioxidants** | | | | **Root antioxidants** | | | |
| --- | --- | --- | --- | --- | --- | --- | --- | --- |
| **Treatment** | **APX** | **CAT** | **POD** | **SOD** | **APX** | **CAT** | **POD** | **SOD** |
| Biochar | 116.7*** | 3016*** | 10238*** | 3274*** | 1053**** | 407.4*** | 456.8*** | 778.5*** |
| Pollutants | 249.2*** | 263.1*** | 1828*** | 598.5**** | 378*** | 707.1*** | 314.3*** | 165.5*** |
| Biochar*pollutants | 5.56** | 5.77** | 22.22*** | 5.44** | 5.84** | 4.71* | 1.58NS | 3.98* |

**Note:** APX=ascorbate peroxidase; CAT=catalase; POD=peroxidase; SOD=superoxide dismutase.

**Table S7 The ANOVA table of redundancy analysis (RDA) between soil properties and leaves antioxidant enzyme activities, photosynthetic parameters, chlorophyll contents, and biomass.**

| Name | Explains % | pseudo-F | P |
| --- | --- | --- | --- |
| Soil pH | 95.8 | 506 | 0.002 |
| CAT | 93.5 | 318 | 0.002 |
| SOD | 87.1 | 149 | 0.002 |
| POD | 82.2 | 102 | 0.002 |
| AK | 79 | 82.7 | 0.002 |
| AP | 77.9 | 77.6 | 0.002 |
| NO_3_^-^-N | 70.4 | 52.4 | 0.002 |
| NH_4_^+^-N | 62.6 | 36.8 | 0.002 |
| CEC | 40 | 14.7 | 0.008 |
| AN | 37.3 | 13.1 | 0.004 |
| APX | 34.5 | 11.6 | 0.004 |

**Note:** APX=ascorbate peroxidase; CAT=catalase; POD=peroxidase; SOD=superoxide dismutase. AP= available Phosphorus, CEC= Cation exchange capacity, AN= available nitrogen and AK= available potassium.

**Table S8 The ANOVA table of redundancy analysis (RDA) between soil properties and root antioxidant enzyme activities, and biomass.**

| Name | Explains % | pseudo-F | P |
| --- | --- | --- | --- |
| NO_3_^-^-N | 80.6 | 91.4 | 0.002 |
| NH_4_^+^-N | 79.1 | 83 | 0.002 |
| SOD | 78.2 | 78.8 | 0.002 |
| APX | 76.5 | 71.7 | 0.002 |
| POD | 70.1 | 51.5 | 0.002 |
| Soil pH | 67.6 | 45.8 | 0.002 |
| CAT | 67.5 | 45.7 | 0.002 |
| AP | 61.1 | 34.5 | 0.002 |
| CEC | 53.9 | 25.7 | 0.002 |
| AN | 49.7 | 21.7 | 0.002 |
| AK | 41 | 15.3 | 0.006 |

**Note:** APX=ascorbate peroxidase; CAT=catalase; POD=peroxidase; SOD=superoxide dismutase. AP= available Phosphorus, CEC= Cation exchange capacity, AN= available nitrogen and AK= available potassium.

**References**

Ahmed, M.B., Zhou, J.L., Ngo, H.H., Guo, W., and Chen, M. (2016). Progress in the preparation and application of modified biochar for improved contaminant removal from water and wastewater. *Bioresource technology* 214**,** 836-851.

Liu, C., and Zhang, H.-X. (2022). Modified-biochar adsorbents (MBAs) for heavy-metal ions adsorption: A critical review. *Journal of Environmental Chemical Engineering* 10**,** 107393.

Shaheen, S.M., Mosa, A., El-Naggar, A., Hossain, M.F., Abdelrahman, H., Niazi, N.K., Shahid, M., Zhang, T., Tsang, Y.F., and Trakal, L. (2022). Manganese oxide-modified biochar: production, characterization and applications for the removal of pollutants from aqueous environments-a review. *Bioresource Technology* 346**,** 126581.

Sun, Y., Shaheen, S.M., Ali, E.F., Abdelrahman, H., Sarkar, B., Song, H., Rinklebe, J., Ren, X., Zhang, Z., and Wang, Q. (2022). Enhancing microplastics biodegradation during composting using livestock manure biochar. *Environmental Pollution* 306**,** 119339.
